# A reliable enzyme-linked immunosorbent assay for the determining of sesame proteins in raw food ingredients and in processed foods

**DOI:** 10.1016/j.fochx.2024.101231

**Published:** 2024-02-15

**Authors:** Masayoshi Tomiki, Masatoshi Sakai, Daichi Tanaka, Mai Hosoya, Kana Uchida, Haruki Shibata, Minoru Morita, Rie Ito, Yusuke Iwasaki, Hiroshi Akiyama

**Affiliations:** a Morinaga Institute of Biological Science, Inc., 2-1-1 Shimosueyoshi, Tsurumi-Ku, Yokohama 230 -8504, Japan; b Department of Analytical Chemistry, School of Pharmacy and Pharmaceutical Sciences, Hoshi University, 2-4-41 Ebara, Shinagawa-Ku, Tokyo 142-8501, Japan; c Division of Foods, National Institute of Health Sciences, 3-25-26 Tonomachi, Kawasaki-ku, Kawasaki 210-9501, Japan

**Keywords:** Food allergen, Extraction, Monoclonal antibody, Sesame, Incurred foods, ELISA

## Abstract

Sesame is a frequent cause of adverse food reactions in allergic patients. We developed a novel sandwich enzyme-linked immunosorbent assay (ELISA) using two monoclonal antibodies and a unique extraction buffer for the detection and quantification of sesame proteins in processed foods and in raw food ingredients to clarify the validity of sesame labeling and for precautionary allergen labeling. The developed sandwich ELISA method is highly specific for sesame proteins. The limit of detection (LOD) and limit of quantification (LOQ) are 0.013 µg/g and 0.025 µg/g, respectively. The recoveries for incurred food samples, such as dressing, breads, sauce and pudding, ranged from 67 % to 81 %, while the repeatability and reproducibility coefficients of variation were less than 4.7 % and 4.5 %, respectively. The developed method has applicability for food products and is a reliable tool for the detection of hidden sesame proteins in raw food ingredients and in processed foods.

## Highlights

• A unique ELISA was developed for sesame protein detection.
• The new ELISA uses two monoclonal antibodies and an original extraction buffer.
• It has high specificity with no cross-reactivity for the studied food materials.
• The new ELISA shows good sensitivity and high recovery.
• It is applicable to investigating hidden sesame protein contaminations in food.

## Graphical abstract

**Figure:**
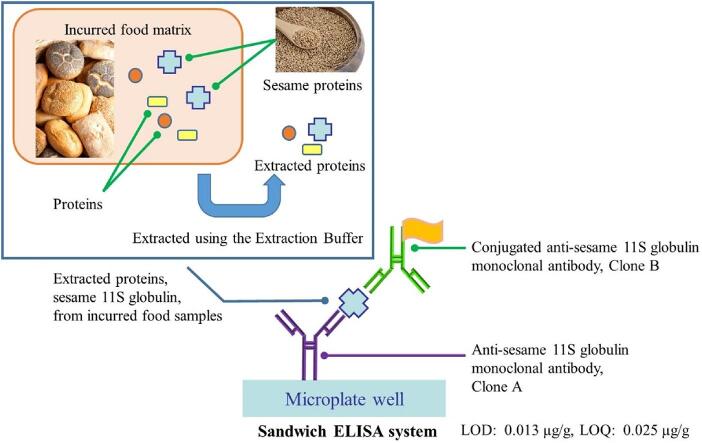

## 1 Introduction

Sesame (*Sesamum indicum* L.) seeds are commonly used in many different types of cuisine, including bakery goods, bread, confectionaries, and as a source of oil. Foods containing sesame are widely consumed in many countries. Sesame can also be a hidden ingredient in many different types of foods, such as sauces, salad dressings, and spice blends. The major global producers of sesame are India, Sudan, Myanmar, China and Tanzania (Wei et al., 2022). The production of sesame seeds in African countries has recently increased, with Tanzania replacing India as the leading producer. Sesame belongs to the genus of *Sesamum* and is a member of the *Pedaliaceae* family. The color of the germplasm allows sesame to be classified as white, black or yellow sesame, with the black and white varieties being more common and widely grown than the yellow variety (Wei et al., 2022).

Sesame seeds are a rich source of protein, with the two major protein fractions being 2S albumin (Ses i 1 and Ses i 2) and 11S globulin (Ses i 6 and Ses i 7). These proteins impart important nutritional and functional properties to sesame seeds and sesame products. 11S Globulin is a storage protein in sesame seeds. Similar to 2S albumin, it is named after its sedimentation coefficient (11S), indicating its size. This protein can cause allergic reactions in susceptible individuals (Beyer et al., 2007).

Sesame is recognized as a major food allergen by Canada, Australia, New Zealand, member countries of the European Union, and Gulf Cooperation Council Standardization Organization member countries (i.e., Kingdom of Saudi Arabia, United Arab Emirates, State of Kuwait, Kingdom of Bahrain, Sultanate of Oman, State of Qatar, and Republic of Yemen) because many people in these countries are allergic to sesame. The population prevalence estimates of sesame allergy range from 0.1 % to 0.2 % in the USA and Canada. In addition, studies report prevalence estimates of 0.1 % in Canadian children and up to 0.8 % among children in Australia (Adatia et al., 2017). Sesame may be added to CODEX, because of the increasing number of patients globally with sesame allergies (FAO & WHO, 2022a). In Japan, sesame is categorized as a recommended labeled food (Akiyama & Adachi, 2021), making it important for food industries to appropriately label sesame in processed foods.

Several detection methods have been developed to confirm the validity of sesame labeling, such as ELISA, RT-PCR and lateral flow devices. Only ELISA methods can determine sesame proteins quantitatively. In 2022, a FAO/WHO expert consultation discussion stated that the threshold for labeling sesame protein be 2 mg/kg (FAO & WHO, 2022b). Appropriate labeling and control precautionary allergen labeling (PAL) for sesame protein requires a methodology specific for determining this protein. An ELISA method developed by Koppelman et al., 2015 can determine sesame protein in foods but may not be applicable to the determination of sesame proteins in processed foods because of low sesame protein recovery from such foods (Koppelman et al., 2015). It is thus important to develop an ELISA method for determining sesame proteins applicable to both raw sesame and processed foods to allow proper ingredient labeling and PAL. The method for determining sesame proteins in processed foods should be reliable and accurate.

In the present study, we developed a unique ELISA method using original extraction solution and two monoclonal antibodies to detect target sesame proteins from both raw food ingredients and highly processed foods to clarify the validity of labeling (Ito et al., 2016). We hypothesized that by using this method, the management of appropriate labeling can be accurately monitored. Furthermore, by making appropriate labeling, it will be possible to avoid allergic symptoms.

## 2 Materials and methods

### 2.1 Food materials

Roasted sesame seeds (white, black and yellow) were purchased from Onizaki Co., Ltd. (Kumamoto, Japan). All sesame seeds were defatted, and roasted white sesame was used as a standard material. The food materials and commercial processed foods were purchased at local supermarkets (Yokohama, Japan).

### 2.2 Chemicals and reagents

Sodium sulfite (Na_2_SO_3_) and sodium dodecyl sulfate (SDS) were supplied by Kanto Chemical Co., Inc. (Tokyo, Japan). Tris(hydroxymethyl)aminomethane hydrochloride (Tris), hydrochloric acid (HCl), polyxyethylene-sorbitan monolaurate (Tween 20), Ethylenediaminetetraacetic acid trisodium salt (EDTA-3Na), 2-mercaptoethanol (2-ME), sodium chloride (NaCl), polyvinylpyrrolidone 25 (PVP25), Sucrose and acetone were supplied by Nacalai Tesque, Inc. (Kyoto, Japan). Bovine serum albumin (BSA) was supplied by FUJIFILM Wako Pure Chemical Corporation (Osaka, Japan), xylitol and methyl alpha-d-glucopyranoside were supplied by Sigma-Aldrich Japan (Tokyo, Japan), horseradish peroxidase (HRP) was supplied by Toyobo Co., Ltd. (Osaka, Japan), Proclin^TM^ 300 (Proclin), 3,3′,5,5′-tetramethylbenzidine (TMB) was supplied by Surmodics, Inc. (Eden Prairie, MN, USA), 4-Hydroxy phenoxy was supplied by Tokyo Chemical Industry Co., Ltd. (Tokyo, Japan) and LIPIDURE®-BL405 was supplied by NOF Corporation (Tokyo, Japan) respectively. Food samples were extracted, and the extracts were diluted, using the extraction buffer solution (Tris-based neutral buffer solution containing 0.1 M sodium sulfite and 0.6 % (v/v) SDS, 0.1 % (w/v) BSA, 0.04 % (w/v) Tween, 200 mM EDTA-3Na and 0.05 % Proclin) and sample diluent (Tris-based neutral buffer solution, 0.1 % (w/v) BSA, 0.04 % (w/v) Tween, 200 mM EDTA-3Na and 0.05 % Proclin). The following buffers were also prepared: 20 mM Tris-HCl (pH 7.4) containing 0.1 M sodium sulfite and 0.6 % (v/v) SDS (buffer A), and 20 mM Tris-HCl (pH 7.4), 150 mM NaCl, 0.05 % Tween 20, 0.1 % 4-Hydroxy phenoxy acetic acid, 0.05 % (v/v) Proclin, 0.125 % (w/v) LIPIDURE®-BL405 containing 1 % (w/v) BSA (buffer B).

### 2.3 Preparation of sesame antibody

Mouse monoclonal antibody IgGs (Clone A and Clone B) were kindly provided by the supplier (the supplier’s name cannot be disclosed due to contract obligations). Clones A and B, against Ses i 6 or Ses i 7, likely recognize the primary structure of the sesame protein 11S globulin using a different epitope for each antibody, given that denatured proteins were recognized by the monoclonal antibodies. However, we cannot verify this due to the supplier’s request for confidentiality.

### 2.4 SDS-polyacrylamide gel electrophoresis (SDS-PAGE)

The sample solution was mixed 1:1 with Laemmli Sample buffer (Bio-Rad Laboratories, Inc., California, USA) containing 5 % 2-ME. The sample was boiled at 100 °C for 5 min and cooled in the flowing water. The sample and a molecular weight marker (SeeBlue Plus2 Prestained Standard, Thermo Fisher Scientific, CA, USA) were applied at 10 μL/lane to the NuPAGE 10 % Bis-Tris Gel 1.0 mm*15well (Thermo Fisher Scientific, CA, USA) in a running buffer (NuPAGE MES SDS Running Buffer (20X), Thermo Fisher Scientific, CA, USA). The electrophoresis was performed at a constant-voltage (200 voltage) for 30 min using an XCell SureLock Mini-Cell apparatus (Thermo Fisher Scientific, CA, USA) under reducing conditions. The gels were stained with Rapid CBB KANTO (Kanto Chemical Co., Inc., Tokyo, Japan).

### 2.5 Western blot analysis using monoclonal antibodies against sesame extracts

The SDS-PAGE procedure was followed by 2.4. The protein was blotted onto a PVDF membrane (Amersham Hybond P 0.45 PVDF, Global Life Sciences Technologies Japan K.K., Tokyo, Japan) in a blotting buffer (a mixture of 10 x Tris/Glycine buffer, pH 8.3 [Bio-Rad Laboratories, Inc., California, USA], methanol and distilled water at 1:2:7) by a blotting system (Trans-Blot SD Cell, Bio-Rad Laboratories, Inc., California, USA). The blotting was performed at a constant electric current (6 mA/cm^2^) for 1 h. The membrane was blocked with a blocking buffer (1 x TBS (50 mM Tris-HCl, 150 mM NaCl) containing 0.1 % BSA and 0.05 % Tween 20) for 1 h at 25 °C. After washing 3 times with a washing buffer (1 x TBS containing 0.05 % Tween 20), the membrane was shaken in a diluted monoclonal antibody (Clone A or Clone B) in the blocking buffer for 1 h at 25 °C. After washing 3 times, the membrane was shaken in a diluted Peroxidase-conjugated AffiniPure Donkey Anti-Mouse IgG (H + L) (Jackson ImmunoResearch LABORATORIES, INC., Pennsylvania, USA) in the blocking buffer for 1 h at 25 °C. After washing 3 times, the membrane was detected with Brown Stain kit (Nacalai Tesque, Inc., Kyoto, Japan). The reaction was stopped by the addition of distilled water.

### 2.6 Sample extraction: spiking incurred foods using white sesame

A Millser IFN-800G homogenizer (Iwatani International Corp., Osaka, Japan) was used several times to completely homogenize the food samples. The extraction buffer solution (19 mL) was added to 1 g of homogenized sample, followed by boiling for 10 min and cooling to room temperature. The pH was confirmed using pH paper to be around 6.0–8.0, then the sample was centrifuged at 3,000 x g for 20 min and the supernatant was filtered through 5A filter paper (Advantec Tokyo Kaisha, Ltd., Tokyo, Japan) to obtain the extract. The sample extract was diluted 20-fold with the sample diluent.

### 2.7 The Kjeldahl method

The Kjeldahl method was performed referring to method 981.10 of AOAC International (AOAC International, 2000). Homogenized sesame seed (0.5 g white, black and yellow) was separately hydrolyzed with 15 mL concentrated sulfuric acid (H_2_SO_4_) containing KELTABS (ACTAC, Tokyo, Japan) and H_2_O_2_ in a heat block at 420 ℃ for 1 h 30 min. After cooling the hydrolysate, H_2_O was added before neutralization and titration. The amount of total nitrogen in each sample was multiplied using the traditional conversion factor of 5.30 to determine the total protein content (Christensen and Fulmer, 1927, Vickery, 1946).

### 2.8 Preparation of calibration standard solution

A calibration standard solution is necessary to quantify sesame protein using the ELISA method. White sesame was ground in a mill and a 50 g aliquot was defatted with 500 mL acetone by stirring for 30 min. The suspension was separated by suction filtration and the pellet was collected. The defatting procedure was repeated three times and the defatted sesame powder was dried for 24 h in the air. Buffer A (20 mL) was added to 0.3 g of defatted sesame powder and the mixture was extracted by boiling for 10 min and cooling to room temperature. The extract was centrifuged at 10,000 x g for 30 min and the supernatant was filtered through a 0.8 µm micro filter paper (DISMIC-25CS, Tokyo Roshi Kaisya Ltd., Tokyo, Japan). The protein content of the initial extract was assayed using a 2D Quant Protein Assay Kit (Cytiva, Tokyo, Japan). The initial extract was diluted to 50 ng/mL using the standard dilution buffer (150 mM Tris-HCl (pH7.4), 0.04 % Tween20, 0.1 % (w/v) BSA, 0.03 % (v/v) SDS, 5 mM sodium sulfite, 2 mM EDTA-3Na and 0.05 % (v/v) Proclin^TM^ 300). The diluted extract was stored at the calibration standard solution for the ELISA. The prepared calibration standard solutions were stored at 4 °C.

### 2.9 ELISA

We referred to the literature method of ELISA described previously (Doi et al., 2008). A micro titer plate (F8 Maxisorp Nucn-Immuno module, Thermo Fisher Scientific K.K., Waltham, MA, USA) was coated at 25 °C for 2 h with the prepared specific monoclonal antibody (Clone A) to anti-11S globulin (100 µL of 6 µg/mL antibody protein solution in 50 mM sodium carbonate (pH 9.6)), then the plate was blocked for 2 h at 25 °C using blocking buffer (20 mM Tris (pH 7.4), 150 mM NaCl, 30 % (w/v) Sucrose, 1 % (w/v) PVP25, 1 % (w/v) BSA and 0.01 % xylitol). The blocking buffer was removed and the plate was dried. The diluted food sample extract and the sesame standard solution were added to the plate (100 µL/well) and incubated for 1 h at 25 °C. After washing six times, the anti-11S globulin antibody (Clone B) labeled with HRP using the literature method described previously (Yoshitake et al., 1982) was diluted as 1 µg/mL with buffer B, added to the ELISA plate at 100 µL/well and allowed to stand for 30 min for the secondary reaction. After washing six times, TMB (100 µL/well) was added to each well and the enzyme reaction was conducted at 25 °C for exactly 20 min. The reaction was stopped by the addition of 100 µL/well of 0.5 M H_2_SO_4_. The absorbance was measured at the dominant wavelength of 450 nm and at 630 nm, the subwavelength. The amount of sesame protein was calculated using the calibration standard curve for sesame protein using the 11S globulin assay as a sesame marker protein. All experiments were performed in duplicate.

### 2.10 Preparation of spiking solution and spiking material

Different sample matrices were spiked with sesame powder to 0.25, 1, 2.5 and 5 ppm for the recovery experiments. Each extraction option was tested using three individual extracted samples per spiked level and the mean value was obtained.

In addition, four different incurred foods were prepared containing 2.5 or 5 ppm sesame protein and tested in the same manner as the spiked food samples.

### 2.11 Preparation of incurred foods

Defatted sesame powder was mixed with the various ingredients shown in Supplementary Table 1 to prepare processed food items containing sesame protein at 2.5 µg/g and 5 µg/g (sesame protein weight/food weight). A detailed recipe of incurred food samples were shown in Supplementary Table 1. The products were stored at −40 °C until use.

### 2.12 Limit of detection (LOD) and limit of quantification (LOQ)

The LOD for the developed ELISA was calculated as 3 times the standard deviation (SD) of the buffer blank mean value after 8 experiments. The LOQ was calculated as 10 times the SD of the buffer blank mean values after 8 experiments (Eidem et al., n.d.).

## 3 Results

### 3.1 Construction of the sandwich ELISA

The target sesame protein for preparation of the antibody was determined by confirming the protein band patterns of the extracted sesame protein solution by SDS-PAGE analysis. Protein band patterns are shown in Supplementary Fig.1, and the 30–32 kDa band was considered as the 11S globulin acidic subunit and the 22 kDa band to be the 11S globulin basic subunit (Onsaard, 2012). 11S Globulin was chosen as the target protein because it was the major protein and was heat-stable in our extraction buffer.

In the Western Blot analysis, the monoclonal antibodies (Clone A and Clone B) reacted to bands with around 32 kDa protein and around 50 kDa proteins in the all sample lanes (Supplementary Fig. 2). These results suggest that the monoclonal antibodies can specifically react with Ses i 6, since the band at around 50 kDa was considered unreduced Ses i 6 whose originally weight is 52 kDa.

To develop a sandwich ELISA for the quantitative detection of sesame in highly denatured processed foods, we used two monoclonal antibodies referred to 2.3. Our previously described unique extraction buffer* was used for sample preparation. The sandwich ELISA system was constructed using two anti-11S globulin monoclonal antibodies that can recognize target proteins processed using the extraction buffer. One monoclonal antibody was used as a capture antibody on a microplate and the other was conjugated with HRP. TMB was used as a substrate.

**Fig. 1:**
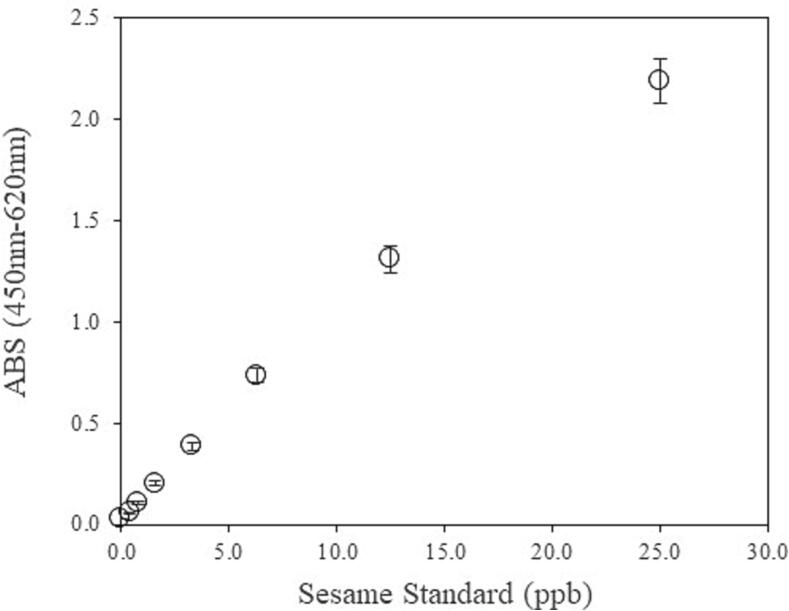
Representative calibration curve of the developed ELISA. The calibration curve was obtained using a four-parameter logistic method using the mean value after eight duplicate analyses. The concentrations of the calibration standard solutions were (µg of sesame protein/g of food weight) = 0.39 ng/mL (0.16 µg/g), 0.78 ng/mL (0.31 µg/g), 1.56 ng/mL (0.63 µg/g), 3.13 ng/mL (1.25 µg/g), 6.25 ng/mL (2.50 µg/g), 12.5 ng/mL (5.0 µg/g) and 25 ng/mL (10 µg/g).

A calibration curve was obtained using the constructed ELISA, as shown in Fig. 1. The values of all calibrators were measured by duplicate analyses. The equation of the calibration curve is y = A + B/(1 + e^[-C - (D*Ln(x)] (x, protein concentration; y, optical density; A = 0.0; B = 14.5; C = -5.5; D = 1.1). The correlation coefficient (r) between the protein concentration and the optical density was determined to be > 0.999. The dose response curves were obtained in the assay. The model that best describes the relationship between the absorbance and concentration of the antigen is a four-parameter logistic curve. The LOD and LOQ were estimated to be 0.013 µg/g and 0.025 µg/g sesame protein, respectively. Consequently, the practical determination range was between 0.39 and 25 ng/mL, representing the minimum level of the standard and the top level of the standard curve, respectively. We constructed the sandwich ELISA using the unique extraction buffer and two monoclonal antibodies to determine the amounts of sesame protein in various extracts.

To demonstrate the extraction system's efficiency, we compared it to PBS (phosphate buffer saline) in a new experiment (Supplementary Table 2). Our original extraction buffer showed significantly higher recovery and better efficiency than PBS.

### 3.2 Reactivity of three kinds of sesame seed and cross-reactivity to food ingredients

White, black and yellow sesame seeds are widely consumed and thus all three kinds of sesame were tested using the constructed ELISA. To compare with the conventional protein assay, we measured the sesame samples using the Kjeldahl method and compared the value with that obtained using the ELISA. Table 1 shows the values obtained using the developed ELISA and the Kjeldahl method. The results suggest that the developed ELISA detects target sesame proteins from all three kinds of sesame seed tested, although the reactivity of yellow sesame was 30 % lower than that of white and black sesame. Therefore, the developed ELISA is applicable to the quantification of all three kinds of sesame seed, and the Kjeldahl method supported the accuracy of the results obtained using the developed ELISA. Given these data and the global consumption of sesame seed, we used white sesame for subsequent experiments such as the validation of matrix effects and the validation of incurred food samples. Thirty-four food ingredients were examined to determine the specificity of the ELISA. As shown in Table 2, all ingredients gave values less than 0.16 ppm, indicating no cross-reaction with major food ingredients such as egg, milk, wheat and soya, suggesting that the developed ELISA has high specificity.

**Table 1:** Reactivity of three kinds of sesame using the developed ELISA.

Sample	Condition	ELISA	Kjeldahl	Ratio[ELISA/Kjeldahl]
		[g of sesame protein/100 g of food]		
White sesame seed	raw	17.2	18.3	94 %
	roasted	19.0	19.2	99 %
Black sesame seed	raw	19.5	17.1	114 %
	roasted	16.7	20.5	81 %
Yellow sesame seed	raw	13.2	15.7	84 %
	roasted	14.0	16.2	87 %

*The extraction ratio is 66.7-fold.

n = 3: one extraction and using three wells.

The sesame extracts were prepared according to the procedure described in section 2.5 of the Materials and Methods. The ELISA mean shows the average value (mg of sesame protein/g of sesame). The Kjeldahl mean shows the average value (mg of sesame protein/g of sesame). The ratio was calculated as ELISA Mean (mg/g)/Kjeldahl Mean (mg/g).

**Table 2:** Cross-reactivity of various food materials using the developed ELISA.

Food samples	Mean*	Food samples	Mean*
Rice powder	< 0.16	Hazel (roasted)	< 0.16
Buckwheat	< 0.16	Almond (roasted)	< 0.16
Wheat	< 0.16	Macadamia (roasted)	< 0.16
Soy	< 0.16	Cashew (roasted)	< 0.16
Peanut	< 0.16	Brazil nut (raw)	< 0.16
Shrimp	< 0.16	Pistachio (roasted)	< 0.16
Crab	< 0.16	Walnut (roasted)	< 0.16
Egg	< 0.16	Pecan nut (roasted)	< 0.16
Egg (boiled)	< 0.16	Sunflower seed (raw)	< 0.16
Dried Egg	< 0.16	Pine nut	< 0.16
Milk	< 0.16	Cumin	< 0.16
Skim milk	< 0.16	Coriander	< 0.16
Corn flour	< 0.16	Black pepper	< 0.16
Lentil	< 0.16	White pepper	< 0.16
Green pea	< 0.16	Turmeric	< 0.16
Lupin beans	< 0.16	Basil	< 0.16
Chickpea	< 0.16	Red pepper	< 0.16

*Mean is the average concentration (µg of sesame protein/g of food sample).

### 3.3 Quantification and validation of the matrix effect using a spiking solution prepared from sesame protein extract

Five matrices (white pepper, dressing, cookie, ice cream and pasta), and water as a blank matrix, were examined to determine their influence on the developed ELISA. As shown in Table 3, the recoveries from all matrices ranged from 81 % to 113 %. The AOAC Appendix M guideline states that the acceptable range of recovery is from 50 % to 150 %. Therefore, we considered that these examined matrices do not affect the results obtained using the developed ELISA and that this ELISA has high accuracy and is not influenced by food matrices.

**Table 3:** Confirmation of several matrix effects using the spiking test with the developed sesame ELISA.

Matrices	Spiked amount(ppm)	Mean(ppm)	Recovery (%)
Water	5.00	5.07	101 %
	2.50	2.72	109 %
	1.00	1.13	113 %
	0.25	0.24	97 %
White pepper	5.00	4.70	94 %
	2.50	2.53	101 %
	1.00	0.95	95 %
	0.25	0.25	99 %
Dressing	5.00	4.70	94 %
	2.50	2.47	99 %
	1.00	0.91	91 %
	0.25	0.23	91 %
Cookie	5.00	5.22	104 %
	2.50	2.54	101 %
	1.00	0.93	93 %
	0.25	0.20	81 %
Ice Cream	5.00	4.40	88 %
	2.50	2.46	98 %
	1.00	0.89	89 %
	0.25	0.21	85 %
Pasta	5.00	5.07	101 %
	2.50	2.70	108 %
	1.00	1.00	100 %
	0.25	0.24	95 %

The indicated amount of sesame protein was spiked into each matrix to confirm the influence of the matrix on the developed ELISA system. The spiked amount (ppm) indicates the concentration in µg of sesame protein/g of matrix. Mean (ppm) is the average concentration in µg of sesame protein/g of spiked matrix. The recovery (%) was calculated as the mean value divided by each spiked amount.

### 3.4 Quantification and validation of sesame incurred food samples

The four tested incurred samples (sauce, dressing, bread and pudding) containing 2.5 ppm or 5 ppm sesame protein (a total of eight incurred food samples) were prepared as per the description in section 2.10 of the Materials and Methods. The incurred foods were evaluated to quantify the sesame proteins using the developed ELISA. Recoveries, the repeatability (RSDr; three independent measurements within a day) and the reproducibility (RSDR; a single measurement on three different days) were evaluated. As shown in Table 4, the mean recoveries for all three incurred food samples ranged from 67 % to 81 %. The RSDr for the eight incurred food samples ranged from 0.5 % to 4.7 %. The RSDR for the eight incurred food samples ranged from 1.9 % to 4.5 %. As both repeatability and reproducibility were below 5 % for all incurred food samples, the results validate that the developed ELISA detects the target sesame proteins from the tested processed foods with high accuracy and precision.

**Table 4:** Recovery, repeatability (RSDr), and reproducibility (RSD_R_) values of the developed sesame ELISA using incurred food samples.

Incurred foods	Mean r (ppm)	Sr	Recovery r	RSDr	Mean _R_ (ppm)	S_R_	Recovery _R_	RSD_R_
Sauce (5 ppm)	3.8	0.18	75 %	4.7 %	3.9	0.14	78 %	3.6 %
Sauce (2.5 ppm)	1.7	0.03	70 %	1.5 %	1.8	0.04	73 %	2.1 %
Dressing (5 ppm)	3.8	0.06	77 %	1.5 %	4.0	0.12	79 %	3.1 %
Dressing (2.5 ppm)	2.0	0.01	79 %	0.5 %	2.0	0.05	80 %	2.7 %
Bread (5 ppm)	3.8	0.05	77 %	1.2 %	4.0	0.08	79 %	2.1 %
Bread (2.5 ppm)	1.7	0.02	67 %	0.9 %	1.8	0.03	70 %	1.9 %
Pudding (5 ppm)	4.1	0.15	81 %	3.7 %	4.1	0.16	81 %	4.0 %
Pudding (2.5 ppm)	1.9	0.06	77 %	3.3 %	1.9	0.09	77 %	4.5 %

Mean r shows the average concentration (µg of sesame/g of incurred food sample) and was used for calculating repeatability. Sr is a repeatability SD (µg of sesame/g of incurred food sample). Recovery r was calculated using the Mean r values and divided by the spiked amount (5 ppm or 2.5 ppm). RSDr was calculated as Sr/Mean r.

Mean _R_ shows the average concentration (µg of sesame/g of incurred food sample) and was used for calculating reproducibility. S_R_ is a repeatability SD (µg of sesame/g of incurred food sample). Recovery _R_ was calculated using the Mean _R_ values and divided by the spiked amount (5 ppm or 2.5 ppm). RSD_R_ was calculated as S_R_/Mean _R_.

### 3.5 Applicability to commercial food products

To examine the applicability of the developed ELISA, thirty-two commercial food samples with or without sesame labeling were analyzed using the developed ELISA. As shown in Table 5, the sixteen commercial foods labeled as containing sesame as an ingredient were appropriately detected above 1.5 µg/g. In contrast, sixteen products lacking sesame labeling on the ingredient list were detected to contain less than 0.16 µg/g (sesame protein/g of food sample weight), which is the lowest detection level on the standard curve. These results suggest that the developed ELISA is applicable for determining sesame proteins in a wide variety of processed foods.

**Table 5:** Applicability of the developed sesame ELISA to commercial foods.

Commercial foods, non-declared sesame	Mean [ppm]	Commercial foods, declared sesame	Mean [ppm]
Bakery	< 0.16	Bakery	>10
Fried noodle	<0.16	Fried noodle	2.51
Beverage	<0.16	Beverage	1.57
Dressing	<0.16	Dressing	>10
Confectionary	<0.16	Confectionary	>10
Snack	<0.16	Snack	>10
Seasoning	<0.16	Seasoning	>10
Freeze-dried food	<0.16	Freeze-dried food	>10
Spread	<0.16	Spread	>10
Miso	<0.16	Miso	>10
Oil	<0.16	Oil	<0.16
Tofu	<0.16	Tofu	>10

The detection of sesame proteins in sixteen kinds of commercial foods comprising both non-declared sesame foods and declared sesame foods. Mean is the average concentration (µg of sesame protein/g of food sample).

## 4 Discussion

We established a sandwich ELISA method using two different monoclonal antibodies and a unique extraction system using SDS/sodium sulfite for the detection of sesame proteins. We showed that the developed ELISA has reactivity to the major sesame varieties regardless of whether the seeds are roasted (Table 1). The results indicate that the protein content in sesame can be accurately determined because the ELISA values are consistent with those obtained using the Kjeldahl method. We found that the amount of protein determined by the developed method in yellow sesame seeds was lower than those in other types of sesame (white and black). The study also suggests that there is no cross-reactivity of the developed method with various food matrices. This is important because cross-reactivity with food materials can cause false-positive results. These results suggest that this method is specific for sesame protein in foods. The LOD of the established ELISA method for a sample solution is 0.013 µg/g. The present results show that the established ELISA method is sensitive and has good accuracy and precision.

An immunoassay must exhibit satisfactory recovery (50 %-150 %) using the AOAC Appendix M (AOAC International, 2012). The present results show that the developed ELISA is suitable for detecting undeclared sesame proteins in both raw materials and processed foods, based on satisfactory recoveries and specificity, although the present validation studies were conducted in-house. Furthermore, we examined thirty-two commercial processed foods, comprising both labeled as containing sesame and no sesame declared, using the developed ELISA. The methodology could detect sesame protein at greater than 10 µg/g in the seventeen tested commercial foods labeled as containing sesame on the ingredients list, and detected sesame protein at concentrations below 0.16 µg/g in the seventeen tested commercial foods not labeled as containing sesame in the ingredients list. These results demonstrated that the developed method would be applicable for processed foods to ensure the validity of the labeling of sesame.

Two immunoassays based on antibodies to sesame proteins have been previously reported (Koppelman et al., 2015, Husain et al., 2010). However, both immunoassays appear to be difficult to apply to processed foods because both studies showed lower recoveries from processed foods due to the use of inadequate extraction systems. We developed the present sandwich ELISA using two monoclonal antibodies and a unique extraction buffer that adequately enables the detection of target proteins from processed foods. We believe that using our unique extraction buffer together with monoclonal antibodies can provide reliable ELISA methods for detecting other allergens such as mustard, cashew nut and walnut.

A further study will require multi-validation to enhance the reliability of the developed ELISA method. Our next investigation will assess various matrices in addition to the current studied matrices.

In conclusion, we developed a reliable, specific and sensitive method for determining sesame protein in both raw ingredients and in processed foods by using our unique extraction buffer and specific monoclonal antibodies. The monoclonal antibodies used in the developed ELISA method can be stably supplied, aiding quality management. The present study demonstrated that the developed method is suitable for the specific quantitative measurement of sesame proteins in processed foods and is little affected by food matrices. The developed ELISA method can be used to accurately monitor the labeling of food products for sesame in a reliable manner and can be useful for the inspections of hidden allergens in food products, confirming our hypothesis. Our developed ELISA can ultimately contribute to avoiding allergic reactions in patients.

## CRediT authorship contribution statement

**Masayoshi Tomiki:** Writing – original draft, Visualization, Project administration, Methodology, Investigation, Funding acquisition, Formal analysis, Data curation, Conceptualization. **Masatoshi Sakai:** Validation, Methodology, Formal analysis, Data curation. **Daichi Tanaka:** Validation, Methodology, Investigation, Data curation. **Mai Hosoya:** Validation, Investigation, Formal analysis, Data curation. **Kana Uchida:** Validation, Investigation, Formal analysis, Data curation. **Haruki Shibata:** Writing – review & editing, Project administration, Funding acquisition, Formal analysis, Data curation, Conceptualization. **Minoru Morita:** Supervision, Resources, Project administration, Methodology, Funding acquisition. **Rie Ito:** Writing – review & editing, Validation, Resources, Formal analysis, Data curation. **Yusuke Iwasaki:** Writing – review & editing, Validation, Methodology, Formal analysis, Data curation. **Hiroshi Akiyama:** Writing – review & editing, Writing – original draft, Supervision, Resources, Project administration, Methodology, Investigation, Funding acquisition, Formal analysis, Data curation, Conceptualization.

## Declaration of competing interest

The authors declare that they have no known competing financial interests or personal relationships that could have appeared to influence the work reported in this paper.

## Data Availability

Data will be made available on request.
