# Supplementary material for: A reliable enzyme-linked immunosorbent assay for the determining of sesame proteins in raw food ingredients and in processed foods
Source: Food Chem X. 2024 Feb 15;21:101231. doi: 10.1016/j.fochx.2024.101231 (PMC10900753; doi:10.1016/j.fochx.2024.101231)
Supplement: Supplementary data 1 [file mmc1.docx]

Supplemental Table 1

Supplemental Table 2

Supplemental Figure 1

Supplemental Figure 2
